# Supplementary material for: The Role of Aesthetics in Intentions to Use Digital Health Interventions
Source: PLOS Digit Health. 2023 Jun 22;2(6):e0000274. doi: 10.1371/journal.pdig.0000274 (PMC10286978; doi:10.1371/journal.pdig.0000274)
Supplement: S1 Text — (DOCX) [file pdig.0000274.s001.docx]

**Pilot Study One**

**Participants**

Twenty participants from the general population were recruited online using posts on social media and research related forums.

**Ethics**

Ethical approval was given by Bournemouth University (ID: 12677).

**Independent Variable**

Ten front page designs based on the facets of the VisAWI that are outlined above.

**Measure**

The VisAWI is a validated 18 item measure of website aesthetics breaks the concept down into four facets. The items of the VisAWI for each facet are outlined below, with reverse scored items highlighted with an (r) mark. Each item is scored on a 7 point likert scale anchored from strongly agree to strongly disagree.

Simplicity

(r) The layout appears too dense.

The layout is easy to grasp.

The layout appears well structured.

(r) The site appears patchy.

Everything goes together on this site.

Diversity

(r) The design is uninteresting.

The layout is inventive.

(r) The design appears uninspired.

The layout appears dynamic.

The layout is pleasantly varied.

Colourfulness

The colour composition is attractive.

(r) The colours do not match.

(r) The choice of colours is botched.

The colours are appealing.

Craftsmanship

The layout appears professionally designed.

(r) The layout is not up to date.

The site is designed with care.

(r) The design of the site lacks a concept.

**Procedure**

Online recruitment material contained a link directing participants to study. Upon accessing the questionnaire participants were presented with a participant information sheet followed by consent.

Once consent was given the participant was directed to the questionnaire which consisted of ten pages, each of which showed a static image of the relevant design followed by the VisAWI items. The order in which the designs were presented, as well as the order of the VisAWI items in each instance, were randomised in order to remove order effects.

**Analysis**

Descriptive statistics were generated for each design in order to compare mean scores for each of the facets of the VisAWI, with differences between facet scores for the positive and negative designs for each facet assessed using paired samples t-test. This has been chosen instead of a repeated measures ANOVA as only the difference in specific facet scores in each instance is of interest rather than the overall difference in scores between stimuli. This does however produce a multiplicity issue, as 25 analyses will be conducted, as such Bonferoni's adjustment has been made resulting in a critical p-value of 0.002 in order to achieve an overall alpha level of 0.05.

**Results**

| Supplementary Table 1: *Table showing the score means and SDs of each VisAWI facet for each of the stimuli designs, as well as the difference and SE between the positive and negative designs for each facet.* | | | | |
| --- | --- | --- | --- | --- |
| **Image** | **Facet - Mean (SD)** | **Image** | **Facet - Mean (SD)** | **Difference (SE)** |
| **Base**  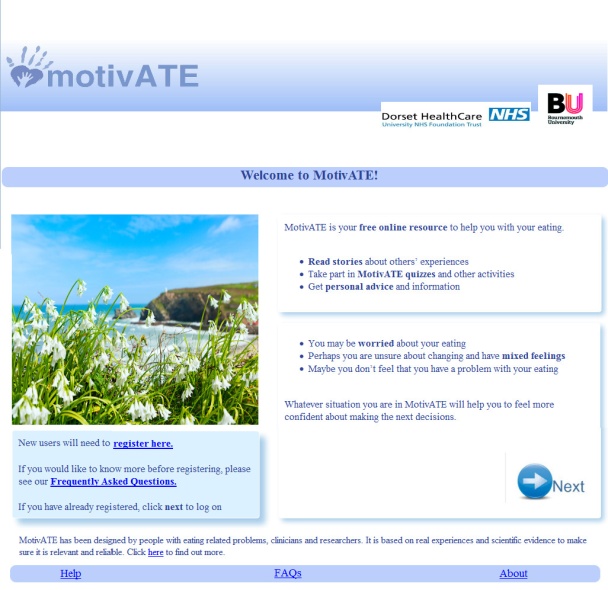 | Simplicity:  4.53 (0.87) | **Full**  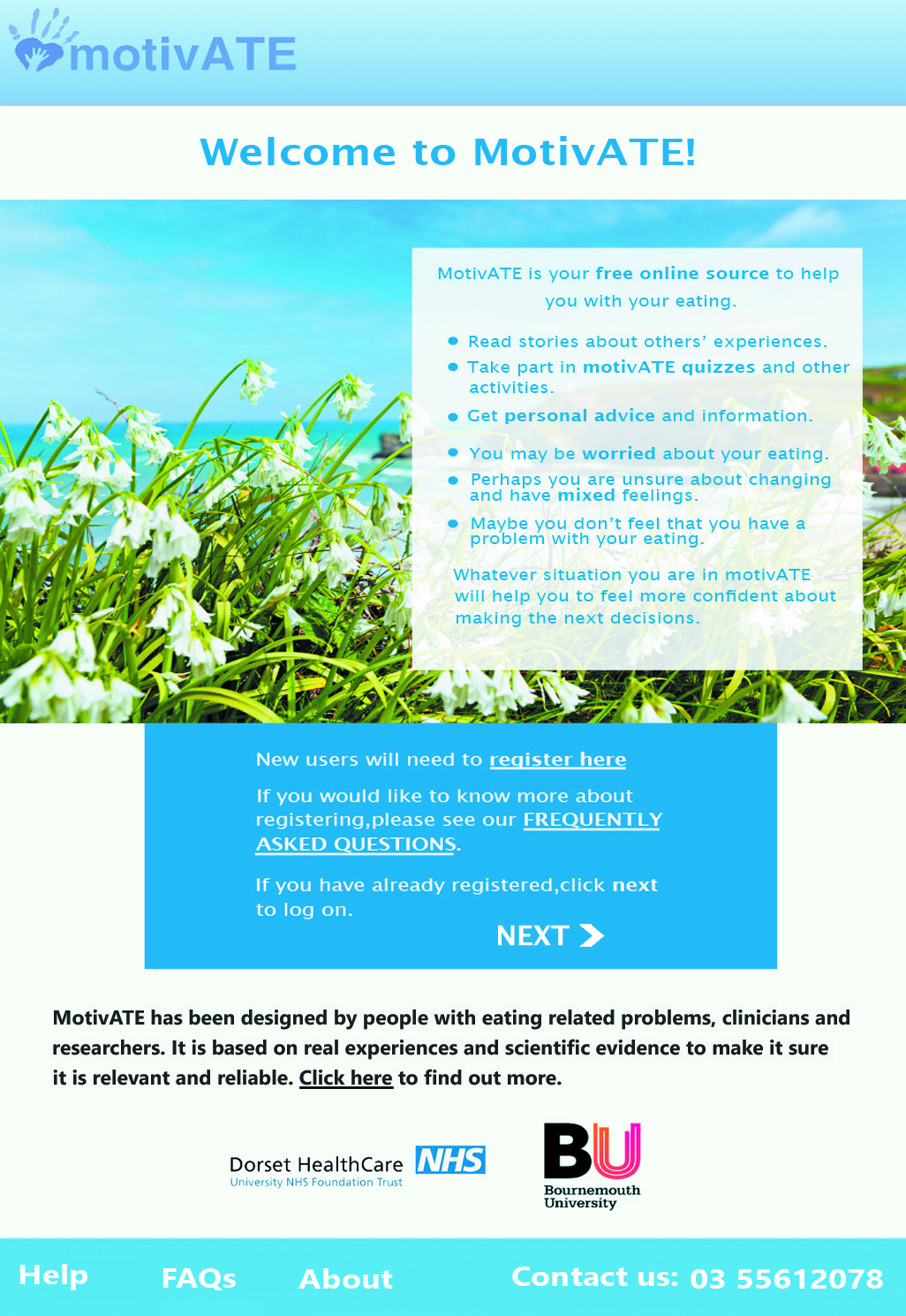 | Simplicity:  5.00 (1.19) | 0.47 (0.33) |
|  | Diversity:  3.39 (1.11) |  | Diversity:  4.56 (1.34) | 1.17 (0.36) |
|  | Colour:  5.04 (1.06) |  | Colour:  5.53 (0.98) | 0.49 (0.27) |
|  | Craftsmanship:  3.6 (1.19) |  | Craftsmanship:  4.71 (1.46) | 1.11 (0.35) |
|  | **Total:**  **4.14 (0.95)** |  | **Total:**  **4.94 (1.15)** | **0.81 (0.31)** |
| **Simplicity Negative**  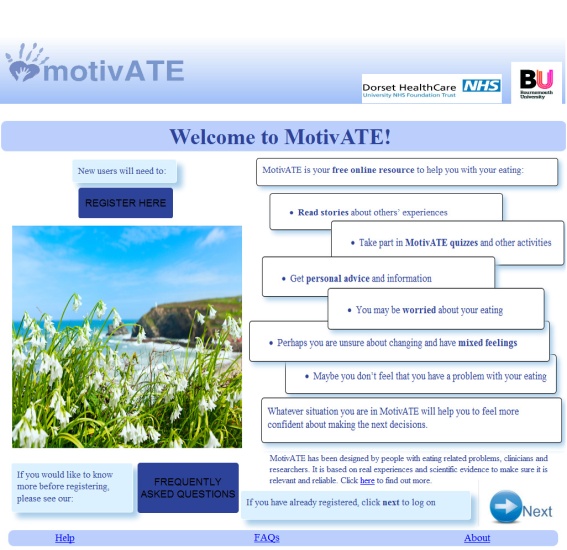 | **Simplicity:**  **1.95 (0.89)** | **Simplicity Positive**  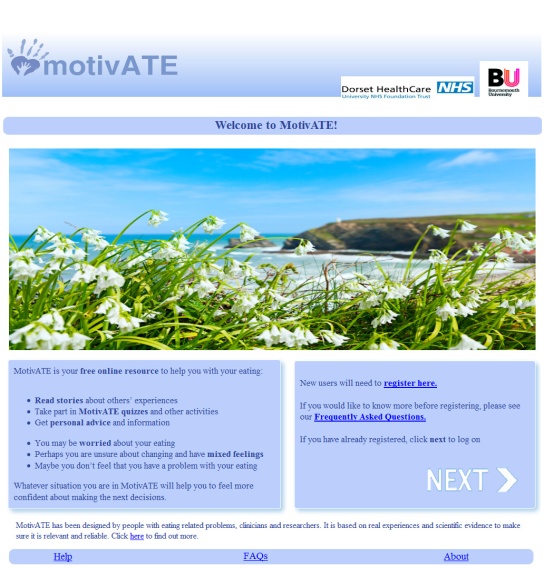 | **Simplicity:**  **5.35 (0.88)** | **3.40 (0.25) **** |
|  | Diversity:  2.82 (1.28) |  | Diversity:  3.80 (1.09) | 0.98 (0.36) |
|  | Colour:  3.98 (1.08) |  | Colour:  5.53 (0.79) | 1.55 (0.24) ** |
|  | Craftsmanship:  2.41 (1.17) |  | Craftsmanship:  4.39 (1.12) | 1.98 (0.31) ** |
|  | Total:  2.79 (0.97) |  | Total:  4.77 (0.78) | 1.98 (0.23) ** |
| **Diversity Negative**  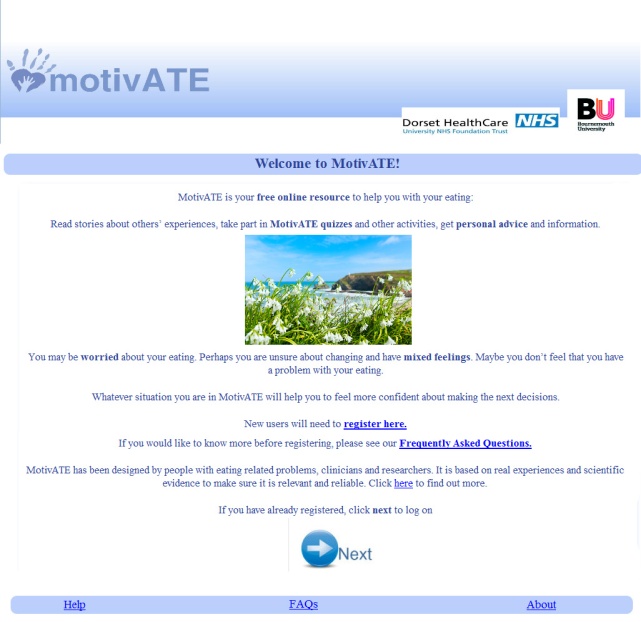 | Simplicity:  3.80 (1.08) | **Diversity Positive**  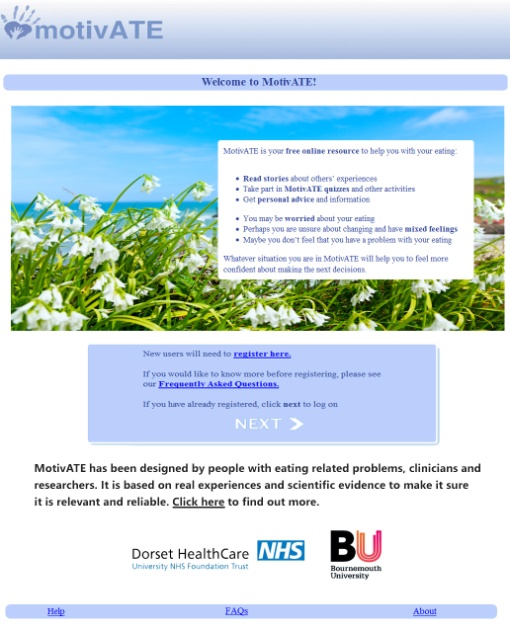 | Simplicity:  4.41 (1.24) | 0.61 (0.27) |
|  | **Diversity:**  **2.24 (1.10)** |  | **Diversity:**  **4.21 (1.50)** | **1.97 (0.42) **** |
|  | Colour:  4.63 (0.99) |  | Colour:  5.11 (1.10) | 0.49 (0.28) |
|  | Craftsmanship:  2.86 (1.19) |  | Craftsmanship:  4.15 (1.39) | 1.29 (0.28) ** |
|  | Total:  3.38 (0.93 |  | Total:  4.47 (1.13) | 1.09 (0.26) ** |
| **Colour Negative**  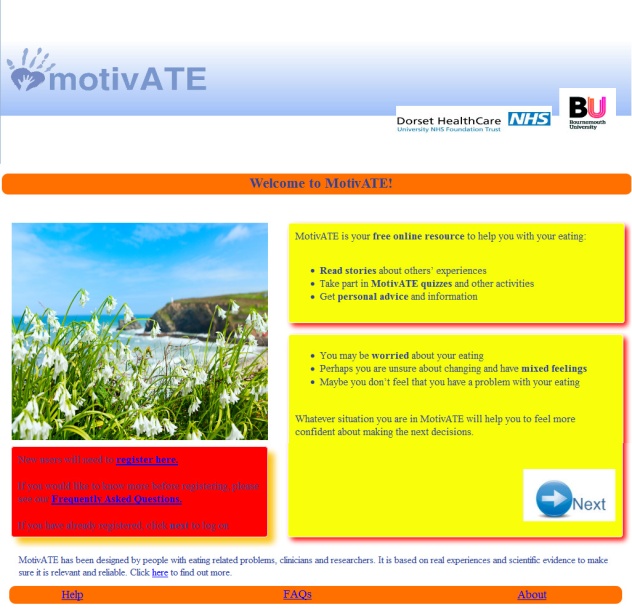 | Simplicity:  3.18 (1.12) | **Colour Positive**  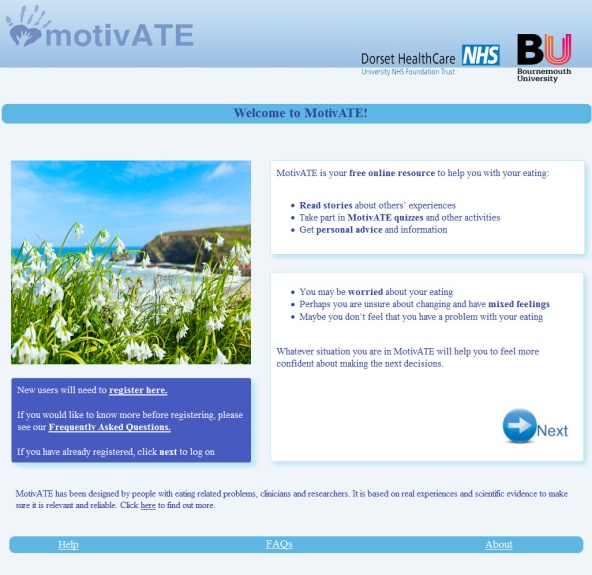 | Simplicity:  4.73 (1.22) | 1.55 (0.39) ** |
|  | Diversity:  2.79 (1.12) |  | Diversity:  3.59 (1.30) | 0.80 (0.30) |
|  | **Colour:**  **1.46 (0.67)** |  | **Colour:**  **5.35 (1.31)** | **3.89 (0.34) **** |
|  | Craftsmanship:  2.43 (0.93) |  | Craftsmanship:  4.10 (1.26) | 1.68 (0.28) ** |
|  | Total:  2.46 (0.85) |  | Total:  4.44 (1.14) | 1.98 (0.27) ** |
| **Craftsmanship Negative**  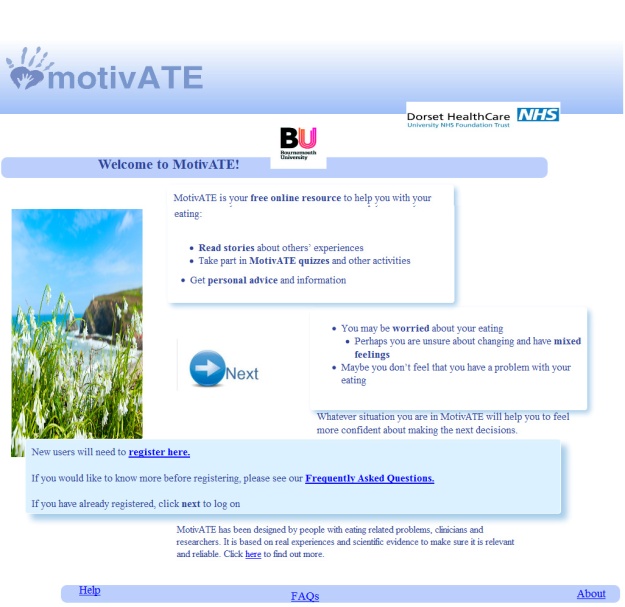 | Simplicity:  2.52 (1.40) | **Craftsmanship Positive**  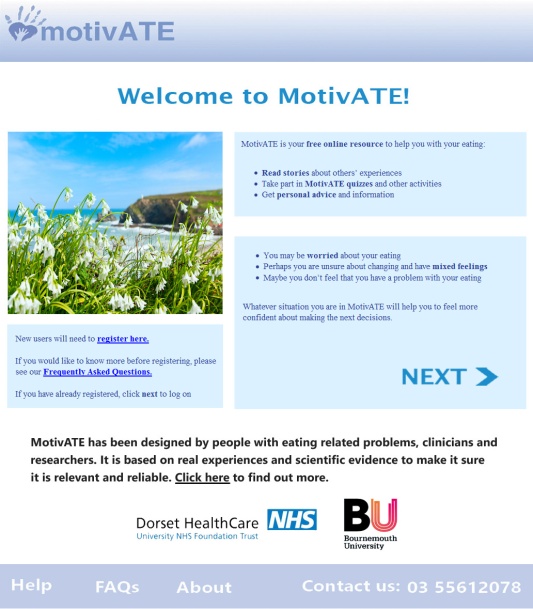 | Simplicity:  4.98 (0.57) | 2.46 (0.28) ** |
|  | Diversity:  2.78 (1.39) |  | Diversity:  3.70 (1.03) | 0.92 (0.27) |
|  | Colour:  4.49 (1.00) |  | Colour:  5.26 (0.69) | 0.78 (0.21) * |
|  | **Craftsmanship:**  **2.21 (1.67)** |  | **Craftsmanship:**  **4.58 (1.08)** | **2.36 (0.41) **** |
|  | Total:  3.00 (1.22) |  | Total:  4.63 (0.70) | 1.63 (0.24) ** |

*Note:* *p < .002, **p < .001

*The target facet for each of the stimuli is highlighted in bold.*

As can be seen from Supplementary Table 1 the results showed highly significant differences between each of the negative and positive stimuli for each facet of the VisAWI on scores for their target facet. However similar differences coud also be seen between most VisAWI facets for each of the stimuli pairs, with the noted exception of the measures between the base and full designs. It is also worth noting that whilst significant, the mean difference between target facet scores for the Diversity and Craftsmanship pairs was markedly smaller than for the Simplicity and Colour pairings. This appears to be a result of lower scores for the positive designs.

**Conclusions**

From this it was concluded that whilst the designs did appear to be successfully targeting the intended facets, improvements were needed for the positive stimuli, particularly those targeting the Diversity and Craftsmanship facets as well as the Full design, in order to produce designs that generated higher VisAWI scores.

**Pilot Study Two**

Following the results of the initial pilot study alterations were made to the Diversity Positive, Craftsmanship Positive and Colour Positive designs. After detailed examination of the results it was decided that the Full design would be dropped from the study, as it did not provide any additional benefits to the study not already provided by the individually targeted positive stimuli. Instead, this design was adapted for use as the Craftsmanship Positive stimuli, as it had scored highest of any of the current stimuli for this facet.

**Ethics**

Ethical approval was given by Bournemouth University (ID: 12826).

**Methods**

The second study replicated the methodology of the first, with the sole difference that in this instance only the adapted designs for the Diversity Positive, Craftsmanship Positive and Colour Positive stimuli were used.

**Results**

| Supplementary Table 2: *Table showing the score means and SDs of each VisAWI facet for the original and updated stimuli designs for Diversity Positive, Craftsmanship Positive and Colour Positive*, *as well as the difference and SE between them.* | | | | |
| --- | --- | --- | --- | --- |
| **Original Stimuli** | | **Updated Stimuli** | |  |
| **Image** | **Facet - Mean (SD)** | **Image** | **Facet - Mean (SD)** | **Difference (SE)** |
| **Diversity Positive**  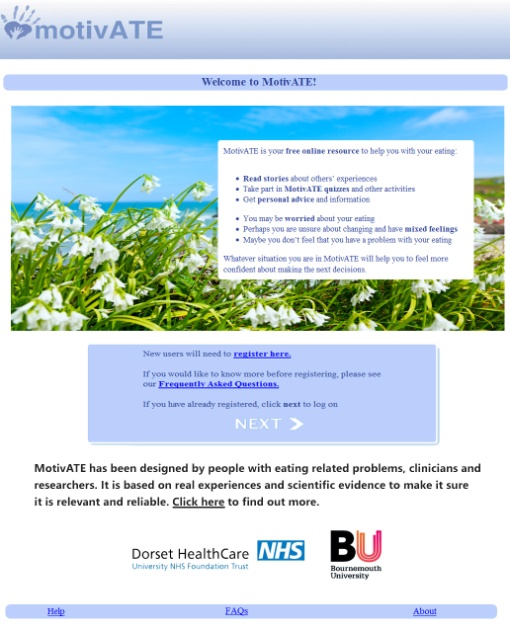 | Simplicity:  4.41 (1.24) | **Diversity Positive**  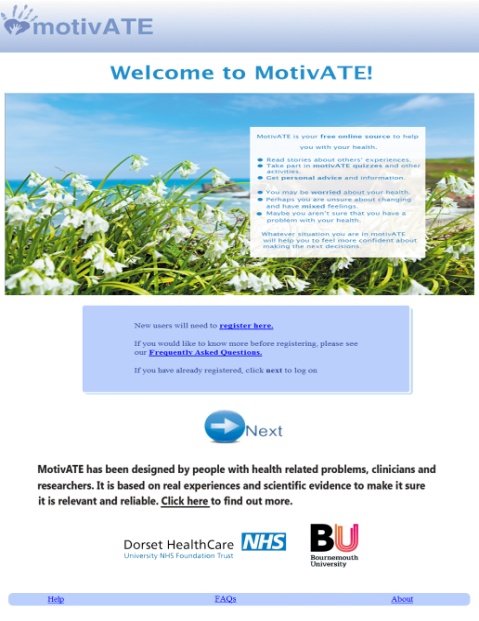 | Simplicity:  4.73 (1.06) | 0.32 (0.37) |
|  | **Diversity:**  **4.21 (1.50)** |  | **Diversity:**  **5.05 (1.25)** | **0.84 (0.44)** |
|  | Colour:  5.11 (1.10) |  | Colour:  5.43 (0.70) | 0.31 (0.29) |
|  | Craftsmanship:  4.15 (1.39) |  | Craftsmanship:  4.45 (0.87) | 0.30 (0.37) |
|  | Total:  4.47 (1.13) |  | Total:  4.91 (0.87) | 0.44 (0.32) |
| **Colour Positive**  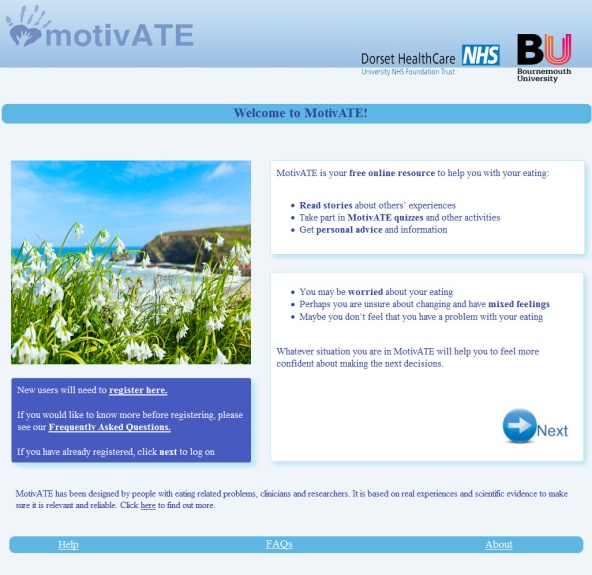 | Simplicity:  4.73 (1.22) | **Colour Positive**  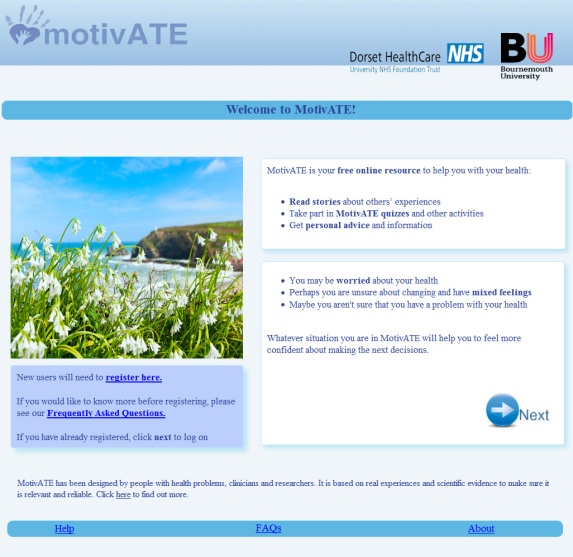 | Simplicity:  4.74 (1.08) | 0.01 (0.36) |
|  | Diversity:  3.59 (1.30) |  | Diversity:  4.11 (1.32) | 0.52 (0.37) |
|  | **Colour:**  **5.35 (1.31)** |  | **Colour:**  **5.90 (0.58)** | **0.55 (0.32)** |
|  | Craftsmanship:  4.10 (1.26) |  | Craftsmanship:  4.23(0.85) | 0.13 (0.34) |
|  | Total:  4.44 (1.14) |  | Total:  4.74 (0.76) | 0.30 (0.31) |
| **Craftsmanship Positive**  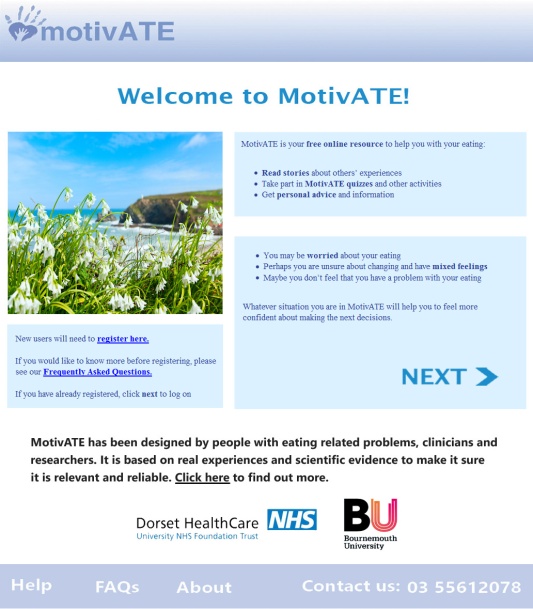 | Simplicity:  4.98 (0.57) | **Craftsmanship Positive**  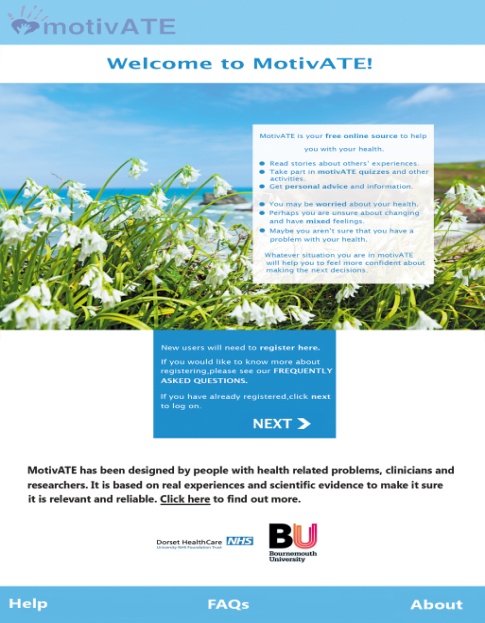 | Simplicity:  4.77 (1.18) | -0.21 (0.29) |
|  | Diversity:  3.70 (1.03) |  | Diversity:  4.63 (1.27) | 0.93 (0.36)* |
|  | Colour:  5.26 (0.69) |  | Colour:  5.54 (0.66) | 0.28 (0.21) |
|  | **Craftsmanship:**  **4.58 (1.08)** |  | **Craftsmanship:**  **5.29 (1.26)** | **0.71 (0.37)** |
|  | Total:  4.63 (0.70) |  | Total:  5.06 (0.97) | 0.43 (0.27) |

*Note:* *p < .05

*The target facet for each of the stimuli is highlighted in bold.*

As can be seen from Supplementary Table 2 the modifications made to the stimuli resulted in small but non-significant improvements to the target facets for each of the stimuli, though in each case a trend towards significance (p<.10) was noted. The only significant change was observed for the diversity facet of the Craftsmanship Positive design. This is unsurprising due to the shift to using the design originally utilised for the Full design, which demonstrated higher scores on this facet during the first round of pilot testing.

**Conclusions**

Whilst the improvements observed were not large enough to achieve significance the updates made to the stimuli were deemed to be sufficient. This is due to both the trend towards significance shown and the fact that the original stimuli already demonstrated significant differences over the negative stimuli for their target facets. As such the purpose of the alterations was simply to produce sufficient improvements in facets scores to elicit a wider range of scores in the final study, which the new stimuli achieved.

**Final Stimuli**

| Supplementary Table 3: Table showing the score means and SDs of each VisAWI facet for each of the final stimuli designs, with updated designs for Diversity Positive, Craftsmanship Positive and Colour Positive, as well as the difference and SE between the positive and negative designs for each facet. | | | | | | |
| --- | --- | --- | --- | --- | --- | --- |
| **Image** | **Facet - Mean (SD)** | **Image** | | **Facet - Mean (SD)** | | **Difference (SE)** |
| **Base**  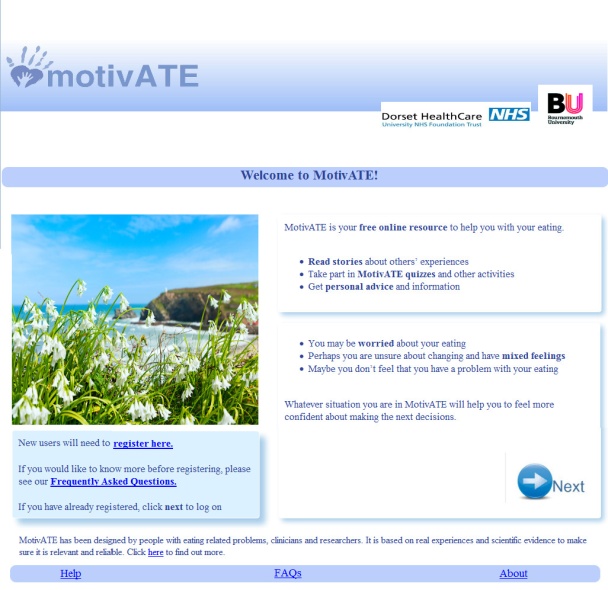 | Simplicity:  4.53 (0.87) |  |  |  |  |  |
|  | Diversity:  3.39 (1.11) |  |  |  |  |  |
|  | Colour:  5.04 (1.06) |  |  |  |  |  |
|  | Craftsmanship:  3.6 (1.19) |  |  |  |  |  |
|  | **Total:**  **4.14 (0.95)** |  |  |  |  |  |
| **Simplicity Negative**  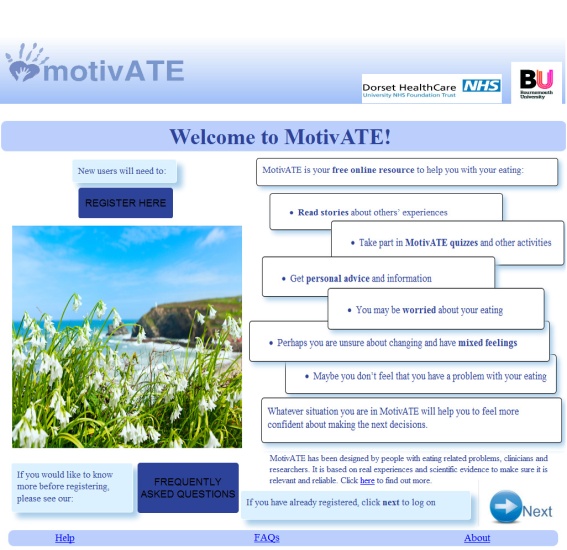 | **Simplicity:**  **1.95 (0.89)** | **Simplicity Positive**  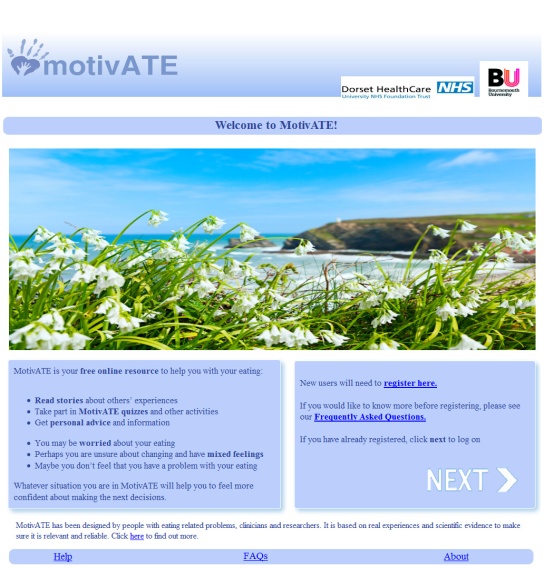 | **Simplicity:**  **5.35 (0.88)** | | **3.40 (0.25) **** | |
|  | Diversity:  2.82 (1.28) |  | Diversity:  3.80 (1.09) | | 0.98 (0.36) | |
|  | Colour:  3.98 (1.08) |  | Colour:  5.53 (0.79) | | 1.55 (0.24) ** | |
|  | Craftsmanship:  2.41 (1.17) |  | Craftsmanship:  4.39 (1.12) | | 1.98 (0.31) ** | |
|  | Total:  2.79 (0.97) |  | Total:  4.77 (0.78) | | 1.98 (0.23) ** | |
| **Diversity Negative**  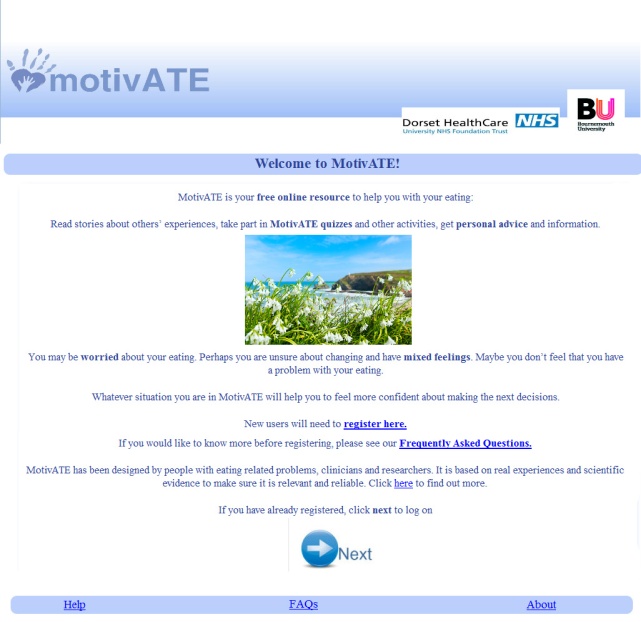 | Simplicity:  3.80 (1.08) | **Diversity Positive**  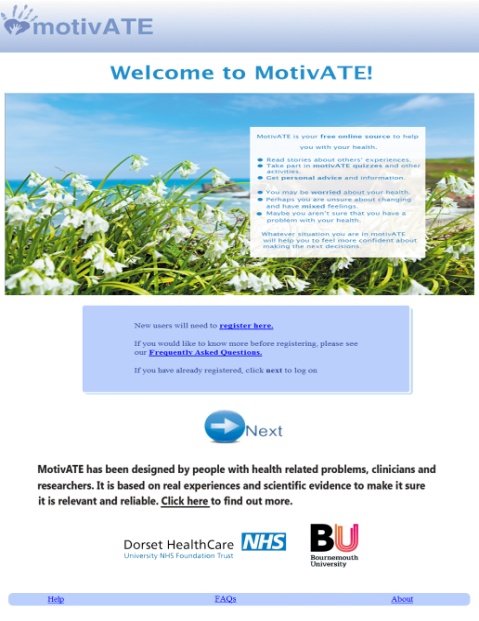 | Simplicity:  4.73 (1.06) | | 0.93 (0.38) | |
|  | **Diversity:**  **2.24 (1.10)** |  | **Diversity:**  **5.05 (1.25)** | | **2.81 (0.34) **** | |
|  | Colour:  4.63 (0.99) |  | Colour:  5.43 (0.70) | | 0.80 (0.29) | |
|  | Craftsmanship:  2.86 (1.19) |  | Craftsmanship:  4.45 (0.87) | | 1.59 (0.34) ** | |
|  | Total:  3.38 (0.93) |  | Total:  4.91 (0.87) | | 1.53 (0.30) ** | |
| **Colour Negative**  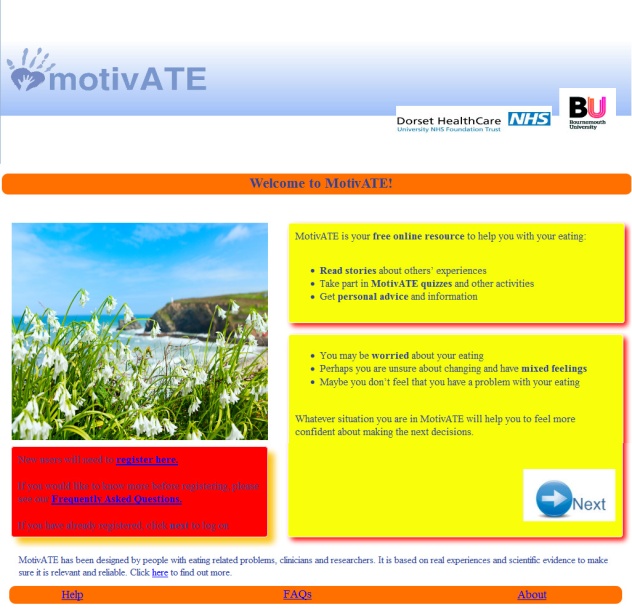 | Simplicity:  3.18 (1.12) | **Colour Positive**  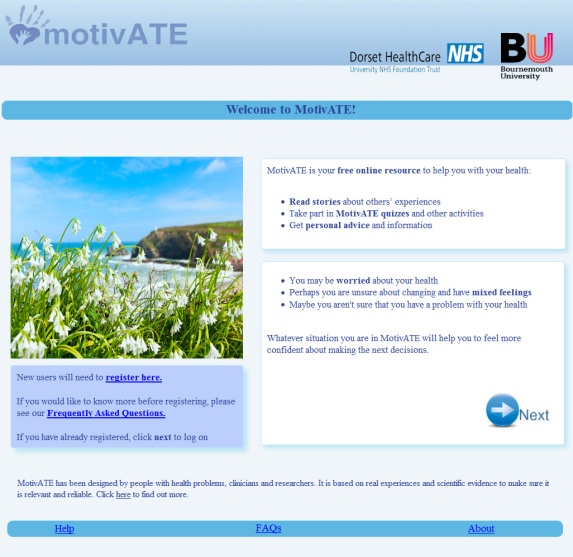 | Simplicity:  4.74 (1.08) | | 1.56 (0.38) ** | |
|  | Diversity:  2.79 (1.12) |  | Diversity:  4.11 (1.32) | | 0.80 (0.40) | |
|  | **Colour:**  **1.46 (0.67)** |  | **Colour:**  **5.90 (0.58)** | | **4.44 (0.22) **** | |
|  | Craftsmanship:  2.43 (0.93) |  | Craftsmanship:  4.23(0.85) | | 1.80 (0.29) ** | |
|  | Total:  2.46 (0.85) |  | Total:  4.74 (0.76) | | 2.28 (0.29) ** | |
| **Craftsmanship Negative**  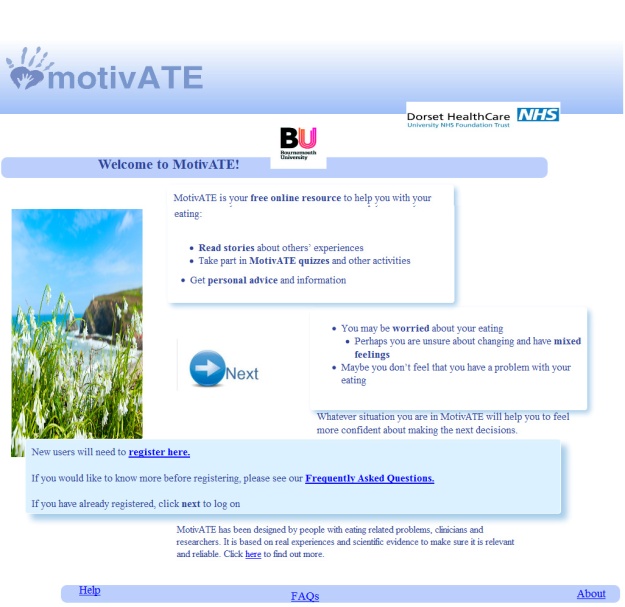 | Simplicity:  2.52 (1.40) | **Craftsmanship Positive**  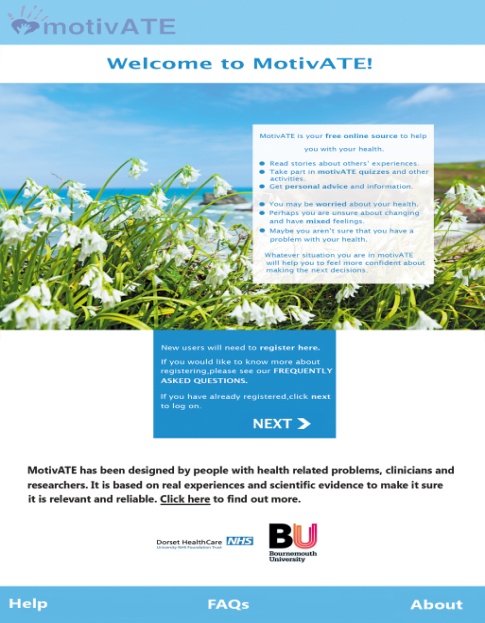 | Simplicity:  4.77 (1.18) | | 2.25 (0.44) ** | |
|  | Diversity:  2.78 (1.39) |  | Diversity:  4.63 (1.27) | | 1.85 (0.46) ** | |
|  | Colour:  4.49 (1.00) |  | Colour:  5.54 (0.66) | | 1.05 (0.30) * | |
|  | **Craftsmanship:**  **2.21 (1.67)** |  | **Craftsmanship:**  **5.29 (1.26)** | | **3.08 (0.44) **** | |
|  | Total:  3.00 (1.22) |  | Total:  5.06 (0.97) | | 2.06 (0.37) ** | |
