# Supplementary material for: The Role of Aesthetics in Intentions to Use Digital Health Interventions
Source: PLOS Digit Health. 2023 Jun 22;2(6):e0000274. doi: 10.1371/journal.pdig.0000274 (PMC10286978; doi:10.1371/journal.pdig.0000274)
Supplement: S2 Text — (DOCX) [file pdig.0000274.s002.docx]

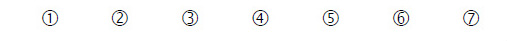

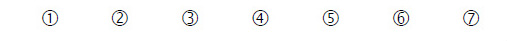

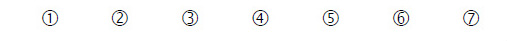

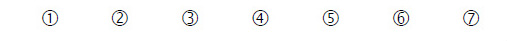

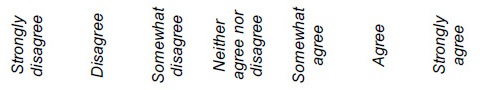


The colour composition is attractive.

The layout appears professionally designed.

The layout is pleasantly varied.

Everything goes together on this site.


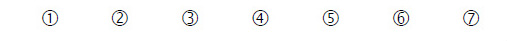

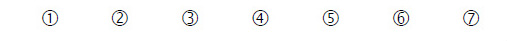

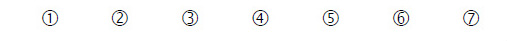

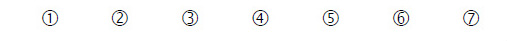

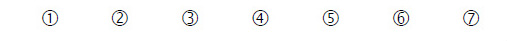

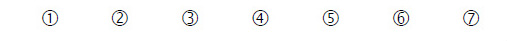

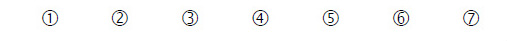

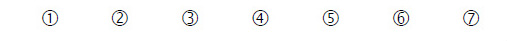

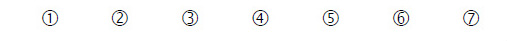

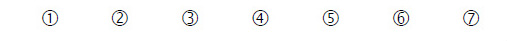

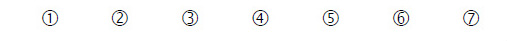

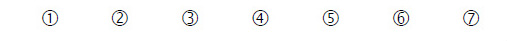


It is likely that I would register with this website.

I feel that this website is trustworthy.

The colours that are used on the site are attractive.

The lay-out of the site is attractive.

I have a positive attitude towards this site.

I think this is a user-friendly site.

The information on this site is interesting to me.

I find this to be a useful website.

I find this website overall an entertaining website.

I intend to visit the site frequently.

I find that this site adds value.

Overall, I find that the website looks attractive.
