# Supplementary material for: The Role of Aesthetics in Intentions to Use Digital Health Interventions
Source: PLOS Digit Health. 2023 Jun 22;2(6):e0000274. doi: 10.1371/journal.pdig.0000274 (PMC10286978; doi:10.1371/journal.pdig.0000274)
Supplement: Attachment — Submitted filename: Reviewer Comments Response.docx [file pdig.0000274.s004.docx]

We would like to thank the reviewers for their detailed and insightful comments on this paper, which have resulted in extensive reworking of both the focus of certain aspects of the manuscript as well as the presentation of the analysis and results. We believe these changes have significantly improved both the focus and clarity of the paper.

Responses to individual comments are made below.

Reviewer #1: Thank you for the opportunity to review your work in, "The Role of Aesthetics in Intentions to Use Digital Health Interventions." I appreciate the need for this research and very much agree with the authors that evidence-based approaches for designing digital health interventions are necessary to increase the likelihood of adoption. This work  also appears to have a potentially informative study design (albeit different than described) and rigorously crafted stimuli that set the authors up well to contribute to the evidence base in this area. However, as written, the methods, in particular the measures and analyses, and findings are not clear, thus, reducing the transparency and potential impact of the work. Below are some comments for consideration.

Abstract
The abstract is the gateway to your work having an impact, and it is clear you have put the time and energy into making it accessible and informative. Very nice! My only minor suggestion is to make explicit the experimental design is within subjects with a repetition among three different populations (see note in methods about this clarification too).

This has been clarified in the abstract.

Introduction
Overall the introduction is well written, making a clear case for the research. I wish the authors had gone with hypotheses instead of research questions, but alas, it it too late for that. Something to consider in future work.

Thank you for this comment, it is something we will strongly consider in future work.

One other thought. Consider cutting the TAM figure. It doesn't add much, and doesn't represent current TAM models (e.g., TAM2, UTAUT). The written argument still holds, as these are still the central pathways of the newer models, but the figure could be removed, which also allows room for more important information (see note below about stimuli).

The TAM figure has been removed, along with in text references to it, as suggested in order to save space for other changes.

Methods
Although this study is described as a "mixed-design," presumably with a 3 level between-persons factors and 9 level within-persons factors, it is not truly a mixed design. Here your between-persons factor isn't a randomized factor, but rather a repetition of the experiment -- albeit with different stimuli? -- among three populations. Please revise to clarify to ensure the study design is not misread.  It is a small clarification in words, but has tremendous impact for how the work can be interpreted. You essentially have three different within-persons studies. This is a valuable design, even if not a mixed design.

Thank you for this very useful clarification, and in hindsight we agree that the method used is not accurately described. This has been updated to describe a quasi-experimental design exploring nine repeated stimuli across three independent groups.

I highly recommend having your final stimuli in the S1 Appendix as a main table. It is fine to keep the process as an Appendix. But without this main table with stimuli, the study is hard to understand. The stimuli is essential for readers. Cut other information to make room (e.g., TAM figure in intro doesn't add much).

Table 3 from appendix S1 showing the finalised stimuli has been included in the methods following the explanation of the stimuli.

It is still not clear how the stimuli content differed for the three populations, the repetition in this research. Please update with more information. This is a critical addition.

Additional explanation of this, including an example, has been added to the methods.

Please include more detail for the number of items for each construct in the methods. This is needed for transparency, potential repeatability of the study, and interpretation of the data. It is not clear what was asked of the participants.

The questions listed in italics along with each construct are the items used to measure it. The text has been updated to make this clearer and an additional appendix (S3) has been added to include a paper copy of the questionnaire to make it easier to understand what questions were asked and how responses were given.

As written, how content relevance could be addressed as a between-persons comparison is not clear. Were participants randomized to see content? It doesn't appear so.

You are correct that they were not, and it is agreed that this was attempting to use the data collected in this research in a way that was beyond its scope. As such this aspect of the paper has been greatly reduced, as it was examining a secondary research question (now removed) that we agree would be more appropriate to explore in a study more explicitly designed to address this question.

The subgroup analyses are first introduced in the data analyses section. These are not well justified. Please address.

The analysis and results sections have been extensively reworked to better introduce, justify and organise the analyses conducted.

Most importantly, the data analysis plan is missing a clear account of what variables were used in what analyses. A MANOVA is not well justified. I do not recommend this approach. In the revisions, I encourage the authors to consider a revised plan that better aligns with the study design. Despite the choices made here, there needs to be a clear account of all predictor and outcome variables for each test conducted.

As this step of the analysis was exploring potential differences in the way participants responded to all the dependent variables between the three conditions in order to assess the best way to combine the data for the final modelling, we feel that a multivariate analysis is the most suitable way to address this. A more detailed justification and explanation for the use of this analysis has been added to the methods. However, we agree that attempts to extend this analysis by examining differences on a univariate level was inappropriate given the study design and has been removed.

Some tests are described in the methods and some in the results. While all findings should go in the results, a clear description with all tests conducted should be in one place -- preferably in the data analysis section of the methods.

The analysis and results have been extensively reworked to provide a clearer account of the analysis conducted in the methods section, and as a result simplify the results to make them easier to follow.

Two phases are described in the data analyses, but more appear in the result. Please revise. None of the models presented in the results are justified or described up to this point.

This has been addressed in the rewriting of the methods and results sections.

Results
Given my confusion about the analyses (see note above), I am struggling to follow the results. Beyond this, the results are generally referred to by the test conducted and not the variables making it even harder to follow. Please revise to also put the variables with whether the findings are significance or not.

A more detailed variable by variable examination of the findings has been added to the results after the final models and diagram.

The abbreviations in the results are tremendously hard to follow in the text. These might work ok for the tables where a key can be provided direct below, but in text it is near impossible to extract meaning without the variable written out.

As part of re-writing the analysis and results sections, use of these terms has been reduced, hopefully making the findings much easier to read.

Significance tests produce a single static number. There can be no trending or other action verb for these results. Please remove. If something is not significant, and by your planned adjusted p-value this would be <=.006, then it is not significant. That doesn't make a finding any less important. Reporting without an action verb does is critical to avoid misleading readers.

This has been removed.

There is inconsistency in the reporting. Some findings were reported as significant at p <.05, which doesn't take into account the adjusted value set up in the methods. Please revise.

This has been revised.

While I was potentially still hanging on Table 4, a series of models looking at a single mediator between aesthetics and behavioral intentions that were newly introduced and not justified, I am completely lost with Table 5. I highly recommend removing these analyses in Table 5, as they do not appear to follow any recommended guidelines for analysis of a within-persons experiment nor are they justified.

This analysis were added both to provide a second, confirmatory, analysis to support the findings of this work, as well as to allow for a more detailed examination of the differences in responses at each step of the likert scale, therefore allowing for a better understanding of where on these scales the most important aspects of design changes lie. However, it is not necessary for our overall conclusions and evidently served to confuse an already complicated process, and as such it has been removed.

Discussion
Unfortunately, because I became so lost in the methods and results,  I am not able to review the discussion. I am unable to interpret the findings to determine whether the discussion aligns with the data or not.

We hope that our rewriting of the previous sections has improved the clarity of our work and as such allow for a proper assessment of the appropriateness of the discussion.

Data
Please update the link to the data. There is currently an error making it so one can't access the data. I did select "yes" above though, as I believe this is an error and not intentional.

Having checked with the repository service used it has come to our attention that they only activate the DOI link provided after receiving proof that associated paper has been accepted for publication. So, the data will become available via this link, but is not currently. Apologies for this oversight, and we would be very happy to provide copies of the data directly to reviewers should they want them.

Reviewer #2: This is a well written study that appears to be rigorously conducted. I don't have expertise in the statistical methods so cannot comment on them but my impression was that the statistical analysis was described well, with justification when required, and presented clearly.

The rationale and methods are clear.

Our thanks for this comment, however as a result of the comments from reviewer #1 this section has been reworked. We hope these are now clearer.

The results are hard to follow in parts due to the complexity of the statistics and the diagram with the Models (A-E) needs a little more description in the figure heading or footnote.

More descriptive detail has been given to this section of the results, and the rational and explanation of the analysis and process of creating this diagram has been expanded on in the analysis section.

The Discussion is appropriate.

Many thanks, we hope the discussion remains appropriate in light of the changes we have made.

The findings are relevant in terms of advancing the field of digital health.

Our thanks for this comment.

I have no further feedback or edits suggested

Thank you again for taking the time to review this manuscript.

Kind regards,

James Denison-Day
